# Syphilis; Its Diagnosis and Treatment

**Published:** 1902-09

**Authors:** 


### Books and Magazines.

---

SYPHILIS; ITS DIAGNOSIS AND TREATMENT. By William S. Gottheil, M. D., Professor of Dermatology and Syphilology, New York School of Clinical Medicine; Dermatologist to the Lebanon and Beth-Israel Hospitals, the West Side German Dispensary, etc. Profusely illustrated. Pages, 216. Price, $1, net. G. P. Engelhard & Company, Chicago, 1901.

This book covers the subject well, bringing the diagnosis and tratment up to date. The author is a clinical teacher, the man above all others who knows best how to set forth the essentials of a subject. T. J. B.

